# Monitoring Essential Health Services During COVID-19 Among Public Primary Healthcare Units in Ethiopia: Existing Capacities and Capacity Gaps

**DOI:** 10.4314/ejhs.v33i2.1S

**Published:** 2023-10

**Authors:** Kassu Ketema Gurmu

**Affiliations:** 1 Health Systems Officer, World Health Organization Country Office, Addis Ababa, Ethiopia; 2 Honorary Assistant Professor, Department of Health Policy and Management, Jimma University, Ethiopia; 3 Adjunct Assistant Professor, Department of Health Policy and Global Health, Addis Continental Institute of Public Health, Ethiopia

COVID-19 has presented all countries and health systems with the challenge to ensure the safe delivery of essential health services. The response to the virus has often led to disruptions to supply chains, shortages of personal protective equipment, reduced and re-purposed staff, and lowered capacity at healthcare facilities (1–4). The diversion of health system resources coupled with public health and social measures to address COVID-19 care has led to a protracted disruption of essential health services, including reduced access to health facilities, reduced ability to pay for services, reduced staff available to provide care, disrupted supply chain systems for essential medical supplies, and lowered service capacity at health care facilities (3,4). At the same time, misinformation has often contributed to changes in health-seeking behavior, and reduced demand for health services. All the above factors have contributed to the disruption in the delivery of health care for all conditions and require systematic and coordinated action to mitigate (3).

Monitoring the continuity of essential health services remains essential not only to mitigate the impact of COVID-19, but also to ensure readiness for other concurrent and future public health emergencies (1). Strengthening primary health care ensures adequate and sustainable quality and distribution of a multidisciplinary workforce, providing high-quality and safe services for both COVID-19 case management and essential health services. Over the course of the pandemic, many valuable adaptations in essential health services have been made to address disruptions. Existing strategies were modified and new strategies have been introduced to address the impacts of the pandemic (5). Ethiopia has taken several progressive measures to control the impact of COVID-19 (6) with several national and international stakeholders including the assistance of the WHO at the national (7) and health facility levels (8).

Countries and health systems need to identify existing capacities and identify gaps and priorities for future interventions to ensure the continuity of essential services during public health and humanitarian emergencies. Thus, studies were undertaken by a multidisciplinary team of experts from four universities (University of Gondar, Jimma University, Hawassa University, and Dire Dawa University) among primary healthcare units across four regions and one city administration in Ethiopia with the objective of identifying the existing availability of services and capacities of primary healthcare units to provide essential health services during COVID-19 and other public health emergencies so as to design priority interventions.

In this special edition of the Ethiopian Journal of Health Sciences, the authors present to readers insightful papers on available capacities and capacity gaps of essential health services (including sexual and reproductive health and gender-based violence services, communicable and non-communicable diseases prevention, diagnosis and treatment services, and maternal, new-born care and child health services) during COVID-19 among primary health care units in Ethiopia as well as demand side factors affecting service utilization. The assessments were done across 452 health facilities (16 hospitals, 92 health centres and 344 health posts) across four regions (Amhara, Oromia, Sidama and Southern Nations, Nationalities and Peoples Region) and one city administration (Dire Dawa Administration) and capacity of primary health care units to provide essential health services during COVID-19. The assessments focused on the six building blocks of the health systems. In addition, a qualitative study on factors contributing to the low utilization of reproductive and maternal health services was undertaken.
